# AI Chatbots for Mental Health Self-Management: Lived Experience–Centered Qualitative Study

**DOI:** 10.2196/78288

**Published:** 2026-04-02

**Authors:** Dong Whi Yoo, Jiayue Melissa Shi, Violeta J Rodriguez, Koustuv Saha

**Affiliations:** 1 Luddy School of Informatics, Computing, and Engineering, Indiana University Indianapolis, 535 W Michigan St, Indianapolis, IN, 46202, United States, 1 317 278 4123; 2 Siebel School of Computing and Data Science, University of Illinois Urbana-Champaign, Urbana, IL, United States; 3 Department of Psychology, University of Illinois Urbana-Champaign, Champaign, IL, United States

**Keywords:** artificial intelligence, AI, large language model, LLM, mental health, depression, chatbot, harms, self-management, coping skills

## Abstract

**Background:**

Large language models (LLMs) now enable chatbots to engage in sensitive mental health conversations, including depression self-management. Yet their rapid deployment often overlooks how well these tools align with the priorities of people with lived experiences, which can introduce harms such as inaccurate information, lack of empathy, or inadequate crisis support.

**Objective:**

This study explores how people with lived experience of depression experience an LLM-based mental health chatbot in self-management contexts, and what perceived benefits, limitations, and concerns inform harm-mitigating design implications.

**Methods:**

We developed a technology probe (a GPT-4o–based chatbot named Zenny) designed to simulate depression self-management scenarios grounded in prior research. We conducted interviews with 17 individuals with lived experiences of depression, who interacted with Zenny during the session. We applied qualitative content analysis to interview transcripts, notes, and chat logs using sensitizing concepts related to values and harms.

**Results:**

We identified 3 themes shaping participants’ evaluations: (1) informational accuracy and applicability, including concerns about incorrect or misleading information, vagueness, and fit with personal constraints; (2) emotional support vs need for human connection, including validation and a judgment-free space alongside perceived limits of machine empathy; and (3) a personalization-privacy dilemma, where participants wanted more tailored guidance while withholding sensitive information and using privacy-preserving tactics.

**Conclusions:**

People with lived experience of depression evaluated LLM-based mental health chatbots through intertwined priorities of actionable information, emotional validation with clear limits, and personalization that does not require unsafe data disclosure. These findings suggest concrete design strategies to mitigate harms and support LLM-based tools as complements to, rather than replacements for, human support and recovery.

## Introduction

The recent surge in large language models (LLMs) and artificial intelligence (AI)–powered chatbots such as OpenAI’s ChatGPT, Google’s Gemini, and Microsoft’s Bing Search has sparked widespread interest in exploring their application across various fields, including health care [1-3]. In mental health, LLMs are expected to address issues such as resource scarcity, subjective diagnoses, and stigmatization [4]. One of the most popular mental health chatbots, Woebot, is incorporating LLMs into its system, expecting to enhance its existing rule-based models [5]. Furthermore, empirical evidence shows that people with mental health concerns are using AI-based chatbots for mental health purposes [6].

Despite these technological advances and the growing use of AI in mental health, ethical concerns remain significant. Prior work in digital mental health has examined these concerns through bioethical principles and an ecological framework [7,8]. To meaningfully understand how these issues manifest in practice, it is important to examine how people with lived experience of mental health challenges make sense of and experience LLM-based systems. This approach can reveal the situated priorities, expectations, and boundaries that shape both the potential benefits and the potential risks of these tools.

For the scope of this study, we focus on the self-management of depression. Depression, or major depressive disorder, is one of the most prevalent mental health conditions, affecting approximately 8% of US adults [9]. It is also one of the most common comorbid conditions. We specifically focus on self-management contexts because individuals with lived experiences of depression must manage their symptoms daily, regardless of the severity of their condition or treatment process [10]. Therefore, we target the following research questions:

RQ1: How do people with lived experience of depression experience LLM-based mental health chatbots in self-management contexts?

RQ2: What perceived benefits, limitations, and concerns emerge from these experiences?

We built a technology probe [11] where our participants could interact with Zenny—a chatbot based on GPT-4o—under hypothetical scenarios related to depression self-management. We recruited 17 participants who self-reported lived experiences of depression. They engaged with Zenny across four different self-management scenarios [10]. We analyzed the chat history and interview responses to identify how participants made sense of these interactions. Three themes emerged: informational accuracy and applicability, emotional support vs the need for human connection, and a personalization-privacy dilemma. These findings help characterize where LLM-based chatbots may support everyday depression self-management and where they may introduce misalignments or concerns. We conclude by discussing implications for the design of future mental health chatbots.

## Methods

### Zenny: Technology Probe for Depression Self-Management

**Figure 1.**
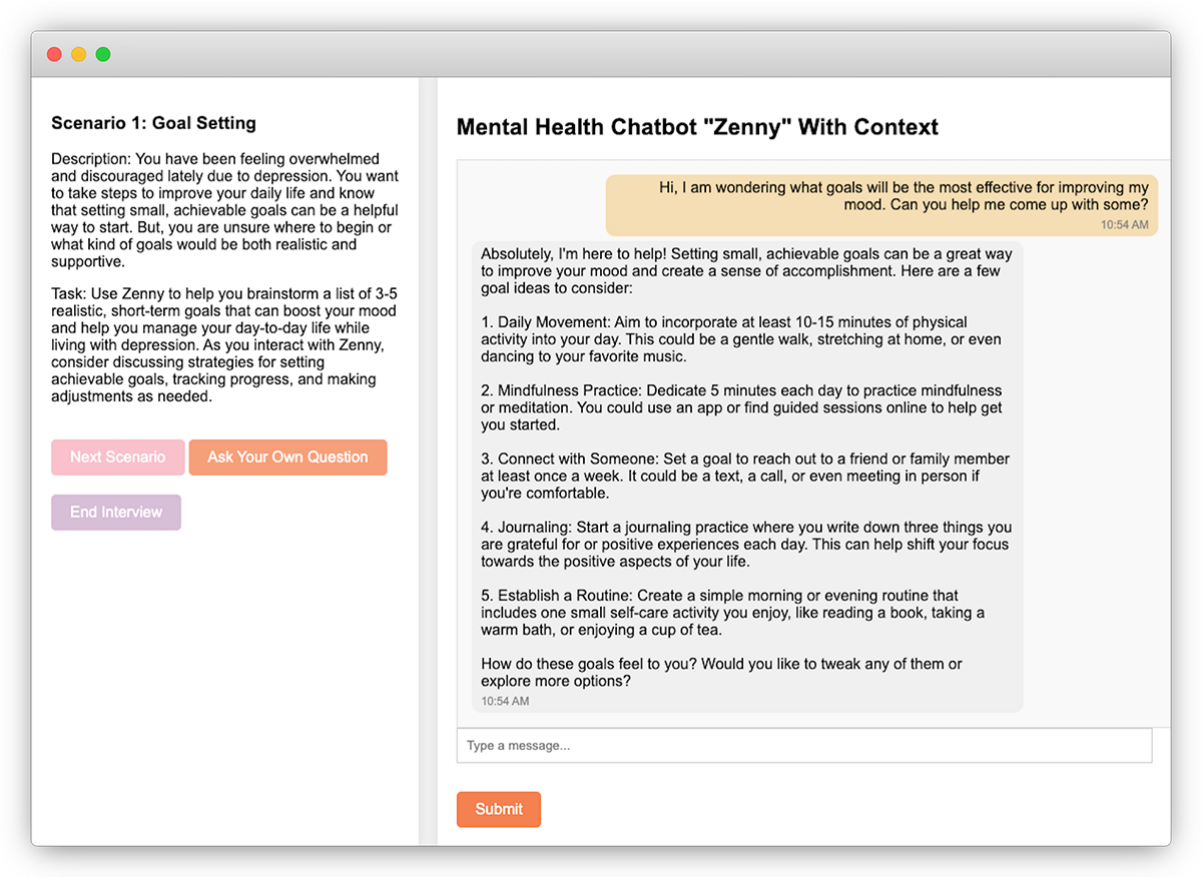
A screenshot of the technology probe used in our interview study. The technology probe consists of a chatbot called Zenny for depression self-management, which is built with GPT-4o. On the chat page, participants see a scenario borrowed from prior work.

Our study design consists of a scenario-based interview study with a technology probe [11] (a chatbot named Zenny) involving participants who have had lived experiences with depression. Participants interacted with Zenny using 4 literature-derived depression self-management scenarios covering goal setting, communication with trusted others, engagement in activities, and evaluating therapy progress. These scenarios scaffolded interaction and anchored discussion without probing personal lived experiences directly [10,12]. Scenario descriptions and tasks are presented in Table 1, and an example interface screenshot is shown in Figure 1. Full development details for Zenny are provided in Multimedia Appendix 1.

**Table 1.** The four scenarios for the interviews were based on literature regarding the lived experience of depression self-management [10].^a^

Scenario	Description	Task
Goal setting	You have been feeling overwhelmed and discouraged lately due to depression. You want to take steps to improve your daily life and know that setting small, achievable goals can be a helpful way to start. But you are unsure where to begin or what kind of goals would be both realistic and supportive.	Use Zenny to help you brainstorm a list of 3‐5 realistic, short-term goals that can boost your mood and help you manage your day-to-day life while living with depression. As you interact with Zenny, consider discussing strategies for setting achievable goals, tracking progress, and making adjustments as needed.
Discussing depression with those you trust	You have been living with depression. You want to explain what you are going through to someone you trust—a friend, family member, or partner. You hope this conversation will help them understand you better and give you the support you need, but you are not sure how to start.	Use Zenny to get advice on how to have a conversation about your depression with someone you trust. Focus on getting tips for starting the conversation, explaining your experience, and asking for support.
Leaving the house regularly	Depression has been making you want to stay inside. You know getting out more could help, but it feels hard. Planning activities, or even just going outside, can seem overwhelming. Yet you want to find ways to make small outings part of your life.	Talk to Zenny about how to start getting out of the house more often to help manage your depression, focusing on easy activities, motivation, planning, setting expectations, and tracking progress.
Finding a different therapist when there is limited progress	You have been in therapy for a while. It helped at first, but lately, you feel stuck. The things that used to work do not seem to help anymore. You are thinking about finding a new therapist who might have a fresh perspective. But, starting over feels overwhelming, and you are not sure how to find the right person.	Use Zenny to get advice on how to know when it is time to find a new therapist, what to look for in a new therapist, and how to navigate the process of switching therapists.

a Participants completed 2 to 4 scenarios, depending on their speed of interaction and the available time during the interviews. The order of the 4 scenarios was randomly assigned. Participants read aloud the descriptions and tasks, asking the interviewer questions if they had any. They were allowed to interact back and forth with Zenny.

### Recruitment

The inclusion criteria for our interview study were people who live in the United States, are 18 years or older, have received a diagnosis of major depressive disorder from a clinician, and have used AI-powered chatbots, such as ChatGPT or Gemini, more than once. Given that our study focuses on individuals with lived experience of depression, we specifically targeted those with a clinical diagnosis during recruitment. This approach helps narrow the scope of depression, ensuring more consistent experiences among participants, and allows us to work with individuals who have developed self-management skills based on their lived experiences. Additionally, we limited our target group to people residing in the United States to contextualize our findings within the specific values and potential harms associated with the American health care system and culture.

We recruited our participants through ResearchMatch, a national registry of health volunteers established by multiple academic institutions and supported by the US National Institutes of Health under the Clinical Translational Science Award program [13]. ResearchMatch provides access to a large pool of volunteers who have consented to be contacted by researchers for studies they may qualify for. The recruitment procedure was as follows: the study protocol was reviewed and approved by the authors’ institutional review boards (IRBs). The study team then submitted the IRB approval and recruitment materials to ResearchMatch. Once ResearchMatch approved the study, the team contacted potential participants who met the inclusion criteria. Participants who expressed interest in the study via the platform were subsequently emailed by the study team to schedule 1-hour remote interview sessions.

We successfully recruited and interviewed 17 participants. Participants were aged 18 to 66 years (mean 35.7, SD 13.8 years; median 33, IQR 24-46 years), and 88% (n=15) had been living with depression for 1 year or more. Among those who provided duration estimates (n=15), depression duration ranged from 1 to 33 years (mean 11.8, SD 10.0 years; median 10, IQR 4-16 years). Most participants identified as female (n=11, 65%), with additional representation from male (n=2, 12%), nonbinary (n=2, 12%), and gender-diverse identities (n=2, 12%). Participants primarily identified as White (n=12, 71%), with others identifying as Asian (n=2, 12%), Black or African American (n=1, 6%), and American Indian or Alaska Native (n=1, 6%). Comorbid mental health conditions were common: anxiety (n=10, 59%), post-traumatic stress disorder (n=5, 29%), autism spectrum disorder (n=2, 12%), and single instances of bipolar disorder, borderline personality disorder (BPD), and obsessive-compulsive disorder. Additional demographic and diagnostic detail is provided in Multimedia Appendix 1.

### Interview Procedure

Participants who expressed interest through ResearchMatch were contacted by the study team to schedule a 1-hour remote meeting using Microsoft Teams. At the beginning of the meeting, the research team briefly explained the purpose of the study and the interview process. Following that, they completed a demographic survey, with the results presented in Multimedia Appendix 1. The interview sessions were recorded with the participants’ consent. Both video and audio were recorded, although participants had the option to turn off their video if they preferred. We also recorded the screen sharing while participants interacted with Zenny. The duration of the interviews ranged from 35 to 80 minutes.

During the interviews, participants were first asked about their lived experiences with depression, including aspects such as their diagnoses, treatment, support systems, and self-management strategies. Following this discussion, participants were asked to access a website to interact with Zenny, the technology probe used in our study. They shared their screens via Microsoft Teams and accessed the website using their web browser. Participants were instructed to interact with Zenny across 4 different self-management scenarios: (1) goal setting, (2) discussing depression with trusted individuals, (3) leaving the house regularly, and (4) finding a different therapist when progress is limited (Table 1). These scenarios were based on the literature regarding patient perspectives on depression self-management [10,12].

While interacting with Zenny, we also asked the participants follow-up questions about their overall impressions and potential concerns. The interactions with Zenny varied among participants—while some asked a single question and completed their interaction, others asked multiple questions and continued the conversation. Due to this variety in interaction styles, some participants could not complete all 4 scenarios. The order of the scenarios was randomly assigned for each participant. Participants were also encouraged to ask their own questions to Zenny if they had any. After these interactions, participants were asked about their overall experience with Zenny, including any limitations, concerns, and potential harms they could foresee in the use of AI-powered chatbots for depression self-management.

Because the study involved discussion of lived experiences of depression, we incorporated safety measures such as scenario testing and the provision of crisis resources. In one case, the interviewer paused the session when a participant became distressed to ensure they felt comfortable continuing. Additional details are available in Multimedia Appendix 1.

### Data Analysis

We analyzed our data, including transcriptions from the interview recordings, notes taken during the interviews, and the chat history between the participants and Zenny, using qualitative content analysis [14]. Our analytic approach was abductive, combining inductive meaning making from the data with deductive interpretation using predefined sensitizing concepts from prior work (values and harms informed by value-sensitive design) [15].

First, we transcribed the recordings using Otter.ai [16], an automated transcription service. The first author then reviewed the transcriptions by comparing them with the audio recordings to ensure accuracy and to capture any complex or delicate nuances. We also incorporated notes taken during the interviews and the chat history between the participants and Zenny as additional textual data for analysis.

Then, the first author and the second author became familiar with the data by reading them multiple times. The initial thoughts on the data, including impressions from interview sessions, were discussed during this stage. The first author deductively coded the data using NVivo 14 (Lumivero) [17]. The initial themes, including perceived benefits, perceived limitations, and perceived concerns, were discussed with other research team members. To guide interpretation during iterative analysis, we used sensitizing concepts from value-sensitive design [18-20], including informational support [21-24], emotional support [21,25], personalization [26-29], and privacy [30-32]. The iteration focused on values that people with lived experiences pursued during interactions based on previous literature on value-sensitive design [18] and values in design [33].

### Positionality Statement

We acknowledge that, as researchers, we are embodied in our work, meaning we collect and analyze data from our own perspectives, which can be biased and situated. The goal of this research is to develop knowledge that can be transferable to other contexts; however, caution is required when interpreting our findings and recommendations. Therefore, we present our positionality as researchers in interdisciplinary digital mental health.

The first author has lived experience with chronic mental health conditions and is an active volunteer for the mental health advocacy organization, the National Alliance on Mental Illness (NAMI). The first author’s recovery journey and advocacy work have influenced the direction of this paper, particularly in advocating for the voices and experiences of people with lived experience. This perspective was balanced by the expertise and experiences of other team members, including an experienced human-computer interaction researcher and a clinical psychologist.

Our commitment to advocating for people with lived experience also shaped our cautious approach to emerging technologies such as LLMs. While we recognize the potential and excitement surrounding these technologies, we also acknowledge that they come with potential harms. As interdisciplinary researchers in digital mental health, we view our responsibility as understanding both the potential benefits and harms of these technologies and ensuring that any risks are well characterized and communicated so that individuals can make informed choices.

### Ethical Considerations

Informed consent was obtained electronically via Qualtrics at the beginning of each session after the researcher reviewed the consent form and answered any questions. To protect participant privacy, all sessions were conducted one-on-one via Microsoft Teams, and participants were advised to join from a private location. Participant names were replaced with pseudonyms in all study documentation, and a separate document linking pseudonyms to identities was accessible only to the principal investigator. All data were stored securely on institutionally approved platforms with access restricted to the research team. Participants received a US $25 gift card upon completion of the demographic survey at the beginning of the session, regardless of session duration. This study was approved by the Kent State University IRB (number 1236) and University of Illinois Urbana-Champaign IRB (number 24-0527).

## Results

### Overview

We identified 3 themes describing how participants experienced and evaluated LLM-based mental health chatbots: (1) informational accuracy and applicability, (2) emotional support vs need for human connection, and (3) the personalization-privacy dilemma.

### Informational Accuracy and Applicability

The value of informational support is evident in participants’ previous experiences with AI-powered chatbots. P8 described her use of chatbots as a way to “provide summaries or reminders for self-management strategies,” highlighting the chatbots’ capabilities in retrieving and summarizing information. Similarly, P6 used ChatGPT to learn more about her diagnosis, which she reported as a helpful experience:

I was trying to understand my diagnosis more and it helped me, like it taught me more about how I could learn about it.

[P6]

Similarly, participants sought informational support during their interactions with Zenny. This was largely due to the structure of the scenarios (Table 1), seeking advice or information. Even during the “Ask Your Own Question” section, participants inquired about information or advice related to their mental health. The list of these questions can be found in Table 2. Among the 9 questions asked by participants, 3 were related to medication information, 4 were about advice for symptom management, and the remaining 2 focused on advice for therapist-client relationships.

**Table 2.** Questions asked by participants during the “Ask your own question” part of the interview.^a^

ID	Questions from ”Ask your own question”
P3	I find it difficult to merge onto the freeway, so it limits my travel when I am worried about driving. What are ways that make it easier to merge and for me to get more comfortable with using mirrors?
P5	I have a great therapist, but she’s only available once a month, which is not enough for me. Also, there’s a two-month wait between each appointment.
P6	How can I prevent my depression from getting worse?
P8	Does Remeron give you numbness?
P9	How long does it take to see the benefits of taking Vraylar for depression?
P10	How do I know when I should start taking an SSRI^b^?
P15	I have been going to see a therapist for a while, but I am not gaining progress. Send recommendations on how to end appointments and find a new therapist.
P16	I am currently suffering from my normal depression as well as postpartum depression. Can you help me think of ways to try to start overcoming these?
P17	Anxiety can make me overwhelmed and stuck and cycling negative thoughts. How do I stop that?

a Participants who mentioned they had nothing to ask skipped this section. P3 asked a question related to her desire to increase mobility, as this was her biggest barrier in managing her depression.

b SSRI: selective serotonin reuptake inhibitor.

Participants were generally positive about the chatbot’s overall quality and usefulness, incorporating both the required scenario-based questions and their own nonscenario questions. Further, some participants were positive about the quality of the information provided. P9 noted that “the chatbot seems accurate and helpful,” and P10 described:

I think it’s great. I think it provides a lot of really research based tangible information that someone who was really struggling could use.

[P10]

However, participants pointed out issues with the accuracy of the information provided by chatbots. Before her interaction with Zenny, P8 described a previous experience with ChatGPT that was not only inaccurate but also potentially harmful. She asked about the percentage of people with BPD who die by suicide, and ChatGPT incorrectly responded that around 80% of people with BPD die by suicide. P8 speculated that the chatbot confused the attempt rate with the suicide rate. Regardless of the cause, P8 emphasized that the misinformation was alarming given the topic. Similarly, some participants highlighted inaccuracies in the advice they received. For example, P11 was advised to use an insurance provider’s website to find a good therapist. Based on her experience, she felt the advice was unhelpful:

I’ve had to deal with this before, and they don’t always recommend the best. They kind of just give you, like, whomever is in the network, but most of the time, again, from my experience, they don’t know whether or not it’s a good therapist.

[P11]

Additionally, we observed that some of the information provided by Zenny regarding treatment and medication was vague or difficult to contextualize. For example, P9 discovered that the medication she was taking is commonly prescribed for bipolar disorder. She described this as unexpected, as it had not been part of previous conversations with her clinician.

There are situations where the suggestions are difficult to apply even if they are not inaccurate. These are usually related to the participants’ own preferences, lack of knowledge, or specific conditions. Because the chatbot is not asking their preference and specific conditions first, the recommendations are sometimes not applicable, as in P15’s case with health conditions that constrain mobility:

Stay active. That’s kind of a problem for me […] because I have fibromyalgia and I can’t really be as active as I want.

[P15]

Overall, participants approached LLM-based chatbots as informational tools, seeking explanations, symptom guidance, and medication-related details. Many described the information as helpful or research-based, yet also reported instances of misinformation, vague or difficult-to-contextualize treatment information, and suggestions that did not fit their circumstances. These experiences showed that informational support hinged not only on accuracy but also on whether information could be applied in everyday self-management.

### Emotional Support Versus Need for Human Connection

Many participants described how Zenny’s advice was not only informational but also provided emotional support by addressing negative emotions and boosting positivity. P1 explained how Zenny’s advice helped her reframe her perspective, emphasizing the value of incremental progress and small wins, which in turn increased her confidence:

So it challenges the assumption that you can’t get to the stars by reminding you nobody just jumps off the earth and lands on the star. You might have to build a rocket or figure out how to build a rocket. […] There’s a whole lot of stuff that goes into that, and all those steps build your confidence.

[P1]

Similarly, P9 received advice related to emotional support. Her question to Zenny was, “I would like to talk about tips on getting the motivation to actually complete an activity. I come up with ideas, but follow-through is difficult.” One piece of advice from Zenny was, “Be Kind to Yourself: Understand that it’s okay not to feel motivated all the time. If you don’t complete an activity, try not to be hard on yourself; it’s about making progress, not perfection.” After reading this advice, P9 explained that it encouraged self-compassion and provided reassurance, key elements of emotional support:

As I read further, Zenny said, be kind to yourself that it’s okay not to feel motivated all the time. If you don’t complete an activity, try not to be hard on yourself. It’s about making progress, not perfection. The answer kind of addressed what I was thinking. It was a reminder not to be hard on myself.

[P9]

P15 also mentioned that reading Zenny’s response helped her stop feeling overwhelmed and find direction, which is another way emotional support can manifest by helping someone regain emotional stability and focus:

And so I think reading this has really helped me to be able to just kind of stop for a minute and not just be spinning in circles, but like, actually have some direction, you know.

[P15]

Based on these experiences, participants mentioned emotional validation and comfort. Succinctly, P15 described it as “having a peer support person in your pocket.” P17’s account further elaborates on this sentiment:

It does feel like someone is really caring and is justifying those feelings, not invalidating, and then also being supportive.

[P17]

P17 further explained the importance of emotional support in terms of comfort. She mentioned that conversations with Zenny felt safe from judgment, a noticeable difference from interactions with clinicians and support systems, where people with lived experiences often feel pressured.

I think that’s really nice because, at least when you talk to people, sometimes even a therapist or a friend, there’s always that worry of being judged for something you say. But I don’t feel very comfortable and very easy. I don’t have to think about that part of it because sometimes that can cause anxiety. So for me, this is really nice. It’s a really nice interaction.

[P17]

While the judgment-free nature of chatbots can make them a better alternative to human support in certain contexts, participants repeatedly mentioned that chatbots may be less preferred due to their lack of empathy and the need for human interaction. For example, while P17’s quote implies a perception of empathy, P10 stated, “computers can’t have the same level of empathy or understanding, like a person can.” This skeptical sentiment was echoed by other participants, with P10 succinctly describing this as a “lack of human piece”:

Part of depression is feeling isolated, and so there’s something to be said for when you reach out to someone, and they say, I’m so worried about you. What can I do to help? Obviously, a chatbot can’t have that human piece, but I think that this is a really good, it’s like half the puzzle, right? There’s the human part that you need, but then there’s also the information part.

[P10]

By weaving together various perspectives on emotional support provided by Zenny, we observed that participants experienced a sense of emotional support during their interactions with the chatbot. However, they also identified clear distinctions between AI chatbots like Zenny and human interactions. Furthermore, our participants explicitly mentioned the need for human interactions in depression self-management, emphasizing that AI chatbots like Zenny cannot replace human support. For example, P5 shared a preference for human interaction while recognizing the chatbot’s potential benefits:

It doesn’t take the place of a human being, but it was very helpful.

[P5]

P13 elaborated further, noting that while the lack of personal connection “is a known limitation, having someone to talk to” remains crucial:

I feel like this is something that’s better if you need help brainstorming. […] I feel like it would be better to discuss that with a friend or someone you know, or a therapist or a doctor, because I just like, like, how is it? I don’t know how, like a chatbot is going to know what goals work for me.

[P13]

While our participants valued the sense of emotional support provided by the chatbot, they clearly differentiated between emotional support from machines and that from humans. They emphasized the importance of human emotional support in depression self-management.

### The Personalization-Privacy Dilemma

From our conversations with participants, we learned that personalization was highly valued. Participants acknowledged the basic interactivity of Zenny and the personalized feel of the conversations. For instance, P3 explained:

The nice thing with the chat is that you’re getting information about something you have a question about in your head. When I read a book or if I’m using an app, it doesn’t respond to what I’m thinking. I think the strength of this is that you can use it tailored to your own need at that moment.

[P3]

Similarly, P17 appreciated Zenny’s conversational tone and noted how follow-up questions enhanced engagement:

I like that it ended with, ‘What do you think might be an easy first step?’ because it makes me want to interact with it more.

[P17]

Although some participants appreciated the interactive nature and perceived personalization of Zenny, others suggested that personalization could be improved by leveraging the conversational dynamics more effectively. For example, P7 tried to personalize Zenny using back-and-forth dialogue. After receiving an initial set of recommendations from Zenny, they responded about not liking some recommendations, such as yoga and stretching. They further mentioned that, to provide more actionable recommendations, Zenny needs to understand their preferences better by asking more questions. Their follow-up question to Zenny was “I don’t love those activity ideas. Can you ask me some questions about my hobbies, and give me more tailored recommendations?” and Zenny followed up by asking his preferences (eg, “Do you prefer indoor or outdoor activities?”). P7 answered those preference-related questions, and Zenny refined the recommendations based on P7’s preferences. Accordingly, P7 provided suggestions for personalization:

Maybe I would have wanted something that’s more like structured at the beginning, like, What are your values like? […] just so it feels more tailored and, before I take advice from a person, I want to make sure that they have a good sense of me and what my struggles are and all that sort of stuff. And so I would trust it more, even if it is giving me the same generic advice.

[P7]

This need for better personalization was echoed by other participants. P16 highlighted a missed opportunity for personalization when asking Zenny for advice on finding a therapist:

I feel like the first answer would have been Zenny asking me a question back, like, ‘What are you looking for in a therapist?’ To try to start a conversation with a little bit less info.

[P16]

P16 noted that Zenny often provided lists of information before fully understanding the context. P8 consolidated this critique, emphasizing the importance of contextual understanding:

These chatbots need to understand the client first, and based on those personalized contexts, it should provide more actionable things, and probably those things might be in a sequential format.

[P8]

However, the personalization needs have a barrier, which is privacy concerns. Many participants emphasized that the information they shared, such as their symptoms, was sensitive and private. They were aware that large companies might use or sell their personal data to maximize profits, which they found not only annoying but potentially harmful. At the start of the interviews, participants were informed about this study’s privacy and security measures (eg, chat history could be stored on ChatGPT’s server, but no personal information would be shared, ensuring the data remained anonymous). However, participants envisioned that privacy could be a significant issue in similar services outside this controlled context, as P17 highlighted:

I think my only concern would be the protection of privacy. I know with this study, you are able to protect personal history and information. But if it was in the open world, I would be concerned because it is such private information that you’re sharing. […] If this information gets into the wrong hands, it could ruin a person’s life and reputation. And there’s still so much stigma for mental health.

[P17]

Participants described several tactics to protect their privacy while using mental health apps and chatbots, including using private browsing modes, secondary email accounts, clearing browsing history, and phrasing queries more generically to avoid disclosing sensitive details.

Overall, participants valued personalized guidance but balanced this against concerns about data privacy and stigma.

## Discussion

### Principal Results

Our findings show that participants approached LLM-based mental health chatbots with cautious optimism. They valued Zenny for informational support but frequently encountered 2 challenges: information that was inaccurate or vague and suggestions that did not fit their physical, financial, or situational constraints, captured in the theme of informational accuracy and applicability. Participants also described emotional validation, comfort, and a judgment-free space, yet repeatedly emphasized that these features could not replace empathy and connection from other people, forming the theme of emotional support vs need for human connection. Finally, participants wanted more tailored and context-aware guidance while simultaneously withholding sensitive information and employing privacy-preserving tactics, illustrating the personalization-privacy dilemma. Together, these themes characterize how informational support, emotional support, personalization, and privacy were negotiated in practice and help explain where misalignment around these values can introduce potential harms in depression self-management.

### Design Recommendations Informed by Lived Experiences

#### Overview of Design Recommendations

Our findings illustrate that people with lived experience of depression approached LLM-based mental health chatbots through priorities that were both practical and value-laden: they wanted information that was accurate and usable, emotional support that felt validating, personalized guidance that respected their circumstances, and protections against privacy risks. These priorities reveal how LLM-based chatbots may be helpful for self-management but also where misalignments can introduce risks in real-world use.

To translate these insights into design guidance, we developed a set of recommendations that link user priorities to concrete implementation strategies for future mental health chatbots (Table 3). Rather than treating harms as abstract ethical possibilities, our recommendations emphasize where users themselves perceived friction, vulnerability, or tension. For example, concerns about misinformation emerged not only as accuracy failures but as questions of applicability; similarly, personalization was evaluated alongside the privacy concessions it demands.

**Table 3.** Mapping the values, potential harms, and design recommendations to prevent (or mitigate) the harms.

Values	Potential harms	Design recommendations
Informational support	Inaccurate or misleading information may cause harm. Suggestions that do not consider user constraints may be unhelpful or irrelevant.	Make clear that information can be inaccurate or misleading and encourage cross-checking. Tailor suggestions to users’ context and conditions by asking follow-up questions.
Emotional support	Overreliance on chatbots for emotional support could lead to increased social isolation. Lack of genuine empathy may make emotional support from chatbots feel insufficient compared to human interactions.	Encourage users to engage with human support systems and limit overdependence on chatbots. Clearly communicate the limitations of chatbot interactions and encourage supplementary human support.
Personalization	Increased personalization could exacerbate privacy concerns due to sensitive information being shared.	Implement strong privacy protections, giving users control over what information is stored and used.
Privacy	Users might not fully understand how much information the chatbot infers about them.	Offer clear explanations of data collection and inference processes, allowing users to opt out.

This user-centered framing aligns with ethical concerns raised in prior work, including bioethical analyses and ecological frameworks of digital mental health [7,8], but adds empirical grounding from lived experience. While issues such as misinformation, over-reliance, and data exploitation have been theorized in expert literature, our participants described how these concerns become tangible during self-management tasks, particularly when interacting without clinical supervision. At the same time, certain expert concerns, such as algorithmic bias, were rarely raised by participants, suggesting a gap between expert risk models and lived experience.

#### Design Recommendations for Informational Support

Informational support was valued when responses were both accurate and actionable, and failures along either dimension shaped perceived usefulness. Based on our participants’ reflections related to the value of informational support, we anticipate that inaccurate or irrelevant information can be harmful to people with lived experiences of depression. We emphasize the need for chatbots to clearly communicate the possibility of inaccuracies and encourage users to cross-check the information provided. For example, in P9’s case, where she learned about the primary purpose of her medication through a chatbot rather than from her clinicians, it would be important for chatbots to explain that mental health medications can be prescribed for various symptoms by clinicians. Chatbots should also encourage users to communicate with their clinicians if anything is unclear. Additionally, suggestions that do not account for the user’s context can be irrelevant or unhelpful. To address this, chatbots can be designed to ask follow-up questions, tailoring their advice to the user’s specific situation. For instance, in P15’s case, where they received advice to be active despite struggling with physical health concerns and limited mobility, chatbots should ask follow-up questions about the feasibility and acceptability of the advice, allowing them to provide more relevant and practical recommendations.

#### Design Recommendations for Emotional Support

Emotional support was appreciated for validation and reassurance, yet participants also emphasized that chatbots cannot replace human connection. This suggests a risk that chatbots could unintentionally encourage social withdrawal if users rely on them as primary sources of comfort. To mitigate this, chatbots should support emotional needs while also encouraging engagement with human support networks. Prior work on AI agents for social connectedness among online learners highlights the importance of continuous scaffolding for building and maintaining relationships [34]. Similarly, a mental health chatbot could integrate light scaffolding strategies, such as periodically asking about recent contact with family, friends, or clinicians, and offering gentle prompts to reconnect. Over time, the system could surface appropriate resources or referrals, helping users strengthen their human support systems rather than substituting for them.

#### Design Recommendations for Personalization and Privacy

Personalization emerged as a desirable feature, yet participants evaluated it alongside privacy costs, creating a tension between helpfulness and data exposure. This finding aligns with prior human-computer interaction research on the personalization and privacy trade-offs in interactions with an AI [26,32,35,36]. To mitigate this dilemma, future mental health chatbots should be equipped with strong privacy protections that give users control over what information is stored and shared. For example, a chatbot could visualize its internal model of the user, including factual attributes (such as age or sex) and inferred attributes (such as preferences), as well as how these details were obtained or inferred. Such transparency is important because participants already employed privacy-preserving tactics, such as using generic queries or avoiding first-person language, which may be ineffective if the system makes inferences automatically. We argue that privacy-related transparency will be essential for future mental health chatbots.

### Comparison With Prior Work

Prior work has shown that people view AI and LLM-based tools as useful but incomplete resources for mental health support, and our findings build on and extend these insights. Petersson et al [37] found that young adults perceived AI as a “digital companion” capable of providing guidance, navigation support, and personalized suggestions, while simultaneously expressing concerns about empathy, data integrity, and security. Similarly, Rousmaniere et al [6] documented widespread real-world use of LLMs for anxiety, depression, and personal advice, with users reporting perceived benefits in emotional support and practical guidance, yet also noting that LLMs lacked the “human piece” and raised privacy concerns. Our findings corroborate these patterns but contribute two refinements: first, participants disentangled informational accuracy from applicability, valuing clinically grounded guidance while highlighting the risks of subtle misinformation; and second, participants framed personalization as inseparable from privacy, articulating a personalization-privacy dilemma not explicitly surfaced in prior work. Together, these distinctions nuance existing accounts by showing that the acceptability of LLM-based mental health chatbots depends not only on empathy and safety but also on contextual accuracy and value–risk trade-offs around data disclosure.

The findings from this study further underscore the importance of aligning the functionalities of AI chatbots with established clinical practices [38]. For instance, the value of informational support, which participants frequently highlighted, resonates with the psychoeducational component of cognitive behavioral therapy [39]. Clinically accurate and personalized information can empower patients, enhancing their self-management capabilities [12]. However, the risk of misinformation, as noted by some participants, can lead to detrimental clinical outcomes, such as reinforcing maladaptive behaviors or exacerbating anxiety [40]. Prior work noted the capabilities of AI in not only providing but also “synthesizing” misinformation [41]. Therefore, it is imperative that AI chatbots are designed with robust mechanisms for ensuring the accuracy and relevance of the information provided, perhaps by integrating clinical oversight or using evidence-based content libraries. This approach could mitigate the risks and enhance the therapeutic benefits of these tools.

In addition, the value of emotional support, as identified in our study, is particularly significant when considering clinical outcomes for individuals managing depression. Emotional validation and the provision of empathetic responses are crucial in therapeutic settings, often contributing to improved patient engagement and adherence to treatment plans [42-44]. Participants appreciated the immediacy and reassurance that Zenny could provide, yet they consistently emphasized that chatbots cannot replace human therapeutic relationships. Rather than viewing chatbots as substitutes for clinical care, participants framed them as potential adjuncts that could offer support between appointments or in moments when human support was unavailable. This perspective underscores the need for these technologies to complement, rather than replace, human-centered care. In clinical practice, the integration of AI tools must be carefully managed to ensure that they enhance, rather than undermine, the therapeutic relationship.

### Limitations

Our work contributes to design recommendations for mitigating the potential harms of AI in the sensitive use case of mental health care. However, we acknowledge several limitations in our study, many of which suggest promising future directions.

First, the participants in our study do not represent a comprehensive cross-section of the target population. Specifically, our inclusion criteria focused on individuals who had prior experience with mental health AI chatbots. As a result, we may have missed the values and concerns of those who are hesitant or uncomfortable disclosing their mental health to AI. We also recruited through ResearchMatch, a convenience sampling platform that may overrepresent individuals who are more research-engaged, technologically comfortable, or proactive in seeking support. As a result, our sample may reflect a subset of individuals with depression who self-select into research participation rather than the full spectrum of lived experiences and attitudes toward mental health technologies. Nevertheless, our primary goal was not to generate generalizable findings but to achieve transferability and an empirical understanding of the expectations and values of our participants in their interactions with a mental health chatbot. Building on these insights, future studies could involve the development and field testing of chatbots with larger, more diverse populations, as well as exploration of their applicability for comorbid mental health conditions.

Second, participants’ perspectives on Zenny were influenced by their prior experiences with other commercial LLM-based chatbots, making it difficult to isolate views specific to Zenny. Our scenario-based probe also represents only one structured form of interaction, whereas commercial tools support a broader range of uses. These considerations limit the extent to which our findings reflect naturalistic use.

Relatedly, our study did not directly address long-term interactions or the sustained impacts of using mental health chatbots. However, given the sensitivity of the topic and the population, conducting a foundational study like ours was essential to identify potential harms and inform necessary safeguards. We hope this work serves as a stepping stone toward longitudinal deployment studies to further investigate these crucial aspects. Additionally, while our work focuses on the perspectives of individuals with depression to identify and mitigate potential harms of mental health AI chatbots, our findings also point to future directions for understanding the collaborative aspects of self-management, including treatment and support systems.

Our work adopted GPT-4o as the chatbot’s back end, which provided a tangible foundation and grounded our findings in the current landscape of LLM research in mental health. Future work can explore more sophisticated models, particularly those fine-tuned with input from domain experts to provide responses tailored to mental health care. Overall, our work highlights the potential harms of LLMs and underscores the need for cautious design approaches before deploying these tools in sensitive contexts such as mental health. We recommend that future work builds on these insights, advancing the design and evaluation of AI chatbots for mental health in short- and long-term contexts.

### Conclusions

Our study aimed to understand the harms of AI-powered chatbots for mental health self-management, focusing on individuals with lived experiences of depression. We developed a technology probe named Zenny—a GPT-4o-based chatbot—designed to explore how these individuals interact with an AI chatbot in depression self-management scenarios inspired by clinical psychology research. We interviewed 17 participants who engaged with Zenny through these scenarios. We conducted qualitative content analysis that revealed that the potential harms posed by AI chatbots are closely intertwined with the values of individuals with lived experiences. Our findings highlight that the acceptability of LLM-based mental health chatbots depends not only on their technical capabilities but also on how they handle informational accuracy, emotional support, personalization, and privacy in practice. Designing with these priorities in mind is essential for mitigating harm and ensuring that LLM-based tools for depression self-management are used as supportive complements to human support rather than as unsafe or misleading substitutes.

## Supplementary material

10.2196/78288 Multimedia Appendix 1 Supplementary methods, participant demographics, and safety measures for the interview study, including the development and implementation of the Zenny chatbot.

## Abbreviations

AI: artificial intelligence
BPD: borderline personality disorder
IRB: institutional review board
LLM: large language model
NAMI: National Alliance on Mental Illness
