# Supplementary material for: AI Chatbots for Mental Health Self-Management: Lived Experience–Centered Qualitative Study
Source: JMIR Ment Health. 2026 Apr 2;13:e78288. doi: 10.2196/78288 (PMC13046095; doi:10.2196/78288)
Supplement: Multimedia Appendix 1 [file mental-v13-e78288-s001.docx]

Supplementary Material

# Methods (Supplementary Information)

## Development of Zenny

To develop Zenny, we used the Flask web framework because of its simplicity and flexibility in web applications. Flask facilitated easy integration with the OpenAI API, which powers Zenny’s conversational capabilities. We developed Zenny using Python virtual environment, and the API key for OpenAI was securely managed. For Zenny’s back-end model, we selected OpenAI’s GPT-4o model because it was the most capable model available at the time of the study. The OpenAI API was incorporated into the Flask application, with careful attention given to constructing messages that define the chatbot’s role, the specific scenario being addressed, and the chat history. The chatbot function includes the scenario’s description, chat history, and fine-tuned user message (e.g., “You are a mental health chatbot named Zenny.”) as inputs. This enabled the model to produce responses that are relevant and aligned with the given scenario.

The front-end design of Zenny features a layout where the left side displays the scenario number, title, description, and task, while the right side is dedicated to participant interactions (Figure 1). All chat history and scenario orders with Zenny were automatically stored on a secured research server, which we later obtained to analyze the chat histories along with the interview transcripts.

## Participant Information

Supplementary Table 1. Demographic information regarding the participants. These abbreviations are used: D (Major Depressive Disorder), A (Generalized Anxiety Disorder), BD (Bipolar Disorders), OCD (Obsessive-Compulsive Disorder), PTSD (Post-Traumatic Stress Disorder), BPD (Borderline Personality Disorder) and ASD (Autistic Spectrum Disorder). These conditions are self-reported by the participants, and all participants confirmed that these diagnoses were made by clinicians.

| **ID** | **Diagnoses** | **MDD Yrs.** | **Age** | **Gender** | **Race** | **Education** | **Occupation** |
| --- | --- | --- | --- | --- | --- | --- | --- |
|  |  |  |  |  |  |  |  |
| P1 | D, A, BD, PTSD | 2 | 53 | Female | White/American Indian | Bachelor’s | Unable to work |
| P2 | D, A, ASD | 7 | 22 | Transgender female | White | High school | Unable to work |
| P3 | D, PTSD | 21 | 46 | Non-binary | White | Bachelor’s | Out of work |
| P4 | D | 2 | 39 | Male | White | Some college, no degree | Employed for wages |
| P5 | D | 30 | 66 | Female | White | Associate | Retired |
| P6 | D | 1 | 18 | Female | Black or African American | Less than high school | A student |
| P7 | D, A, ASD | 10 | 23 | Male | White | Bachelor’s | Out of work |
| P8 | D, BPD | 10 | 22 | Genderqueer female | White | Master’s | Out of work |
| P9 | D, A, PTSD | 33 | 53 | Female | White | Some college, no degree | Unable to work |
| P10 | D, A, PTSD | - | 48 | Female | White | Master’s | Employed for wages |
| P11 | D, A | 13 | 25 | Female | American Indian or Alaska Native | Master’s | A student |
| P12 | D, A | 12 | 28 | Female | White | Master’s | Employed for wages |
| P13 | D, A | - | 29 | Female | Asian | Master’s | Employed for wages |
| P14 | D | 8 | 24 | Female | White | Bachelor’s | A student |
| P15 | D, A, OCD, PTSD | 3 | 42 | Non-binary | White | Master’s | Self-employed |
| P16 | D | 20 | 36 | Female | White | Associate | Out of work |
| P17 | D, A | 5 | 33 | Female | Asian | Master’s | Employed for wages |

## Safety Measures for the Interview Process

Due to the sensitive nature of the lived experience of depression, we implemented multiple safety measures for the interview process. Our research team included human–computer interaction researchers with experience in mental health, as well as a clinical psychologist. The research team discussed the potential risks of this interview study and collaborated on safety measures.

Accordingly, we developed the technology probe/chatbot (Zenny) with careful consideration of the safety of the participants. The scenarios were drawn from the literature of previously empirically validated themes of depression self-management [1,2], in consultation with our clinical psychologist coauthor. Before conducting the participant interviews, we rigorously tested the responses to the several query permutations corresponding to the scenarios to ensure the chatbot did not generate problematic responses.

To support participants who might experience distress during or after the interview, we provided a list of mental health crisis support resources in the consent form, including the 988 Suicide and Crisis Lifeline (https://988lifeline.org), 7 Cups of Tea (https://7cupsoftea.com), and Crisis Text Line (https://crisistextline.org).

During the study, one participant became agitated while discussing difficult emotions related to family members. The interviewer paused the session to check if they felt comfortable continuing, and they were reminded of the available mental

health resources. No other participant reported experiencing distress after the interviews.

**References**

1. Van Grieken RA, Kirkenier ACE, Koeter MWJ, Nabitz UW, Schene AH. Patients’ perspective on self‐management in the recovery from depression. Health Expectations 2015 Oct;18(5):1339–1348. doi: 10.1111/hex.12112

2. Van Grieken RA, Van Tricht MJ, Koeter MWJ, Van Den Brink W, Schene AH. The use and helpfulness of self-management strategies for depression: The experiences of patients. Withers MH, editor. PLoS ONE 2018 Oct 25;13(10):e0206262. doi: 10.1371/journal.pone.0206262
